# Computational analysis of pathogen-borne metallo β-lactamases reveals discriminating structural features between B1 types

**DOI:** 10.1186/1756-0500-5-96

**Published:** 2012-02-14

**Authors:** Eithon Cadag, Elizabeth Vitalis, Kristin P Lennox, Carol L Ecale Zhou, Adam T Zemla

**Affiliations:** 1 Global Security Computing Applications Division, Lawrence Livermore National Laboratory, Livermore, 94550 CA, USA; 2 Biosciences & Biotechnology Division, Lawrence Livermore National Laboratory, Livermore, 94550 CA, USA; 3 National Security Engineering Division, Lawrence Livermore National Laboratory, Livermore, 94550 CA, USA

## Abstract

**Background:**

Genes conferring antibiotic resistance to groups of bacterial pathogens are cause for considerable concern, as many once-reliable antibiotics continue to see a reduction in efficacy. The recent discovery of the metallo β-lactamase *blaNDM-1 *gene, which appears to grant antibiotic resistance to a variety of Enterobacteriaceae *via *a mobile plasmid, is one example of this distressing trend. The following work describes a computational analysis of pathogen-borne MBLs that focuses on the structural aspects of characterized proteins.

**Results:**

Using both sequence and structural analyses, we examine residues and structural features specific to various pathogen-borne MBL types. This analysis identifies a linker region within MBL-like folds that may act as a discriminating structural feature between these proteins, and specifically resistance-associated acquirable MBLs. Recently released crystal structures of the newly emerged NDM-1 protein were aligned against related MBL structures using a variety of global and local structural alignment methods, and the overall fold conformation is examined for structural conservation. Conservation appears to be present in most areas of the protein, yet is strikingly absent within a linker region, making NDM-1 unique with respect to a linker-based classification scheme. Variability analysis of the NDM-1 crystal structure highlights unique residues in key regions as well as identifying several characteristics shared with other transferable MBLs.

**Conclusions:**

A discriminating linker region identified in MBL proteins is highlighted and examined in the context of NDM-1 and primarily three other MBL types: IMP-1, VIM-2 and ccrA. The presence of an unusual linker region variant and uncommon amino acid composition at specific structurally important sites may help to explain the unusually broad kinetic profile of NDM-1 and may aid in directing research attention to areas of this protein, and possibly other MBLs, that may be targeted for inactivation or attenuation of enzymatic activity.

## Background

Proteins within the β-lactamase family have long drawn the attention of researchers and clinicians due to their ability to efficiently hydrolyze many common antibiotics. Metallo β-lactamases (MBLs) in particular are of global health interest, as many are acquired, capable of traveling across species, and are the most commonly encountered transferable carbapenemases [1]. The recently discovered plasmid-borne New Delhi metallo β-lactamase (NDM-1), capable of hydrolyzing a broad range of antibiotics, is such a metalloenzyme and is noted for its ability to confer resistance to all but a small handful of β-lactam antimicrobials. First characterized within a Swedish patient of Indian origin in 2008 [2], NDM-1 has since been identified in other parts of Asia, North America, Europe, Australia and Africa [3-8].

In addition to its rapid worldwide dissemination, NDM-1 is alarming for its penchant to transfer between species *via *conjugation. With its initial identification on a 180-kb *Klebsiella pneumoniae *plasmid, and subsequent re-discovery on a *Escherichia coli *plasmid isolated from the same patient, NDM-1 has displayed an ability to spread amongst bacteria [2], and more recent findings have identified it in additional members of the Enterobacteriaceae family [9,10]. Moreover, the presence of the gene encoding NDM-1 within isolates has been associated with the presence of genes and genetic elements which confer additional resistance against other forms of antibiotics, including monobactams, aminoglycosides, fluoroquinolones and tetracyclines [5,9,11], further reducing treatment options for infected patients.

Taken within this context, NDM-1 has the potential to greatly impact global health, most immediately in hospital settings through nosocomial infections, which appear to be a common mode of infection for NDM-1 carrying bacteria [4]. Further knowledge of the mechanisms of the encoded protein may help to expedite development of therapies and countermeasures. Preliminary characterization of NDM-1 conducted by Yong and colleagues revealed marginal sequence similarity to other members of the MBL family, with the closest sequence homology to VIM-1 and VIM-2 at only 32%; kinetic studies supported this association, although NDM-1 was noted to possess a superior binding profile for most β-lactams compared to VIM-type proteins [2]. They further identified, using sequence alignment, novel features of the NDM-1 protein not found in other members of the MBL family, such as uncommon residues around the zinc binding site and a four-residue insertion not observed in other MBLs. These features may help provide NDM-1 with its capability to readily bind to a very broad range of β-lactams. Other, more well-known MBLs such as the VIM-type proteins found in Pseudomonas, have likewise spread rapidly since their initial discoveries [12-15]. Many infections have been transmitted nosocomially, and are often found in developing areas [4,16]. Further knowledge of the mechanisms of these metalloenzymes may help to expedite development of inhibitors with direct clinical significance.

The variety, structure, function and medical significance of these proteins have been the focus of much research in the past, and they may be classified both molecularly and functionally. Traditionally, MBL proteins are categorized as "class B" β-lactamases, which can be further divided into subclasses based on the nature of the metal binding site. The presence of specific binding motifs around the active cavity of the proteins, associated with zinc binding and coordination, may be used to classify an MBL as either B1 (zinc binding at H116-H118-H196 and at D120-C221-H263), B2 (N116-H118-H196; D120-C221-H263) or B3 (H/G116-H118-H196; D120-H121-H263) [17]. Notably, four of the six conserved residues are static across all classes, allowing amino acid-based molecular classification at only two positions (H/N/G116 and C221/H121). This classification scheme, though simple, is thought to be strongly related to the structural plasticity of the enzymes, as the zinc binding sites are critical to the hydrolytic effects of MBLs. Functional groupings have also been used as a means of describing similarities between MBLs. Inhibition by EDTA, substrate hydrolysis rates and profiles created by testing against other inhibitors (*e.g.*, clavulanic acid) can be used to profile clinically relevant groups of MBL proteins and identify isolates in the lab [18,19].

Prior research on the structure-function relationship of MBL proteins has focused primarily on the region of the active site and mechanism of catalysis. For di-zinc MBLs, hydrolysis is believed to occur *via *breaking of the β-lactam amide bond on the carbonyl by a resident hydroxide in the active site. This action is zinc-activated, and creates a temporary intermediate tetrahedral carbon, upon which the zinc-bound water donates a proton to the leaving nitrogen of the ligand [20-23]. The steps involved in this action are believed to be ligand-dependent, and protonation may or may not coincide with cleavage of the β-lactam ring (*e.g.*, as noted for nitrocefin bound to ccrA) [24]. For B1 MBLs, binding is thought to be mediated in part by the presence of a large mobile flap that forms a cleft over the active site [21]. Deletion of this flap region in some MBLs has been correlated with weakened affinity for many antibiotic substrates, with the exception of imipenem [25]. The mobile flap exists in B1 MBL types ccrA and IMP-1 with an aromatic, bulky residue, and has been hypothesized to be critically involved in the recruitment, stabilization and binding of inhibitors [21,26,27]. This flap is less functionally important in VIM-2, which contains an alanine (A64) in place of an aromatic residue [28], exemplifying the nuanced structural functionality of common B1 MBL components.

The prevalence of methods for classifying MBLs is in large part due to their functional, structural and molecular similarities and differences, and our work builds upon the features used for classification currently receiving attention by applying structure-based analyses of well-characterized MBLs, with the hope of identifying residues and regions that can further aid in functional discrimination. A more detailed picture of residue conservation and structural uniqueness is assembled for proteins within the B1 MBL subclass, and its constituent types VIM, IMP, ccrA and NDM-1. While the core structure of MBLs is well known to be conserved, structural alignments revealed a "linker" region with considerable variability among B1 proteins, which we propose as a notable structural classification feature. We apply structural analysis methods to the NDM-1 protein in order to identify significant sequence and structural differences from other MBLs that may affect NDM-1's ability to bind to antibiotics. Recently solved crystal structures of the NDM-1 protein [23,29,30] are compared with B1 subfamilies IMP-, ccrA- and VIM-type structures for the purposes of identifying distinctive features. Using structure-based sequence variability analyses and clustering of key features, structurally conserved residues were identified in NDM-1 and compared with the corresponding residues in similar proteins to identify regions of conservation and novelty between the known and new B1 MBLs. Many sites we identify computationally as highly conserved correspond to those found to be functionally critical by prior experimental work. Common themes, as well as features unique to NDM-1, are identified. Of particular interest is an uncharacteristically divergent "linker" region. We find that while the vast majority of B1 MBLs' conformation is well conserved, NDM-1 is marked by both the presence of rare residues in resistance-implicated regions and a linker conformation that is unique among MBL structures.

## Methods

### MBL-like protein structure library

As one goal of this study was an overall structural characterization and comparison of available B1 MBL structures, with emphasis on the recently discovered NDM-1 protein, a library of B1 MBL proteins for which both sequence and structure were available was generated. Protein structures were retrieved from the Protein Data Bank (PDB) [31], and full sequences were taken from UniProt [10]. Special focus was given to three specific B1 types used in comparison to NDM-1 from prior research: IMP-1, VIM-2 and ccrA [2,23,29,30].

**Figure 1:**
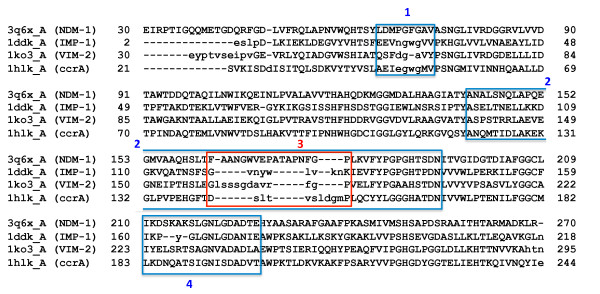
**Structure-based sequence alignment of NDM-1, IMP-1, VIM-2 and ccrA**. Alignments were generated using LGA with NDM-1 (3q6x_A) as the reference. Capitalized amino acids indicate structural residue-residue correspondence, whereas lowercase amino acids indicate regions in MBLs where no structural alignment with NDM-1 can be identified. Regions of interested are denoted by boxes and labeled as the mobile flap (1, blue) (residues 65-73 in NDM-1; 23-31 in IMP-1; 59-67 in VIM-2; and 44-52 in ccrA), the linker region including anchoring regions (2, blue) (residues 141-193 in NDM-1; 98-143 in IMP-1; 138-200 in VIM-2; and 120-166 in ccrA), the non-aligning linker region core (3, red) (residues 163-179 in NDM-1; 120-129 in IMP-1; 174-186 in VIM-2; and 142-152 in ccrA), and the L10 loop (4, blue) (residues 210-227 in NDM-1; 160-174 in IMP-1; 223-240 in VIM-2; and 183-200 in ccrA). Notably, ccrA is the only B1 MBL gap free with respect to NDM-1 outside of the linker region.

Representatives of IMP-1 (PDB: 1ddk_A), VIM-2 (PDB: 1ko3_A), ccrA (PDB:1hlk_A) and NDM-1 (PDB: 3q6x_A) were selected as seed structures for expanding the number of structures used for variability analysis (see 2.2) to include similar, MBL and MBL-like, proteins; these representatives are used as the reference structures for their respective types throughout the rest of our study, unless otherwise noted. Figure 1 shows structure-based sequence alignment between the selected representatives, showing strong overall correspondence with relatively few gaps. Structure-based similarity searches were performed for IMP-1, VIM-2, ccrA and NDM-1 against the entire PDB database (release 2011/08/02; 188,448 chains) using the StralSV algorithm [32]. Pruning of the retrieved structures was performed via an LGA_S [33] cutoff value of ≥ 50% structure similarity to the corresponding reference structures. After removing PDB chains identical in sequence, the result consisted of 75 structures. This set of proteins was expanded to include all available NDM-1 crystal structures (nine, as of the writing of this manuscript), which formed the final MBL fold library (83 structures; refer to Additional file 1) used for comparative computational analysis.

### Comparative structural analysis

Members of the MBL-like library were subjected to a number of comparative methods in order to determine distinctive regions of conservation and divergence. Structure-based sequence variability analyses were run for the representative structures of NDM-1, IMP-1, VIM-2 and ccrA, using StralSV [32], which calculates sequence variability from fragment-based local structural alignment. The purpose of this analysis was to identify in analyzed MBL structures local regions where proteins are structurally unique, and regions where they are relatively conserved regardless of their sequence similarity, focusing on sequence compositions in such regions.

The StralSV algorithm works, briefly, as follows: a target structure, *t*, and associated library, *L*, are specified. Template structure *l *∈ *L *shares structural similarity with *t *in at least some structural fragments. Detection of local similarities and calculations of alignments between *t *and all *l *are performed using the LGA program [33], and the specific residue-residue correspondences for *t *and each member of *L *are found. Thus, for each position in *t*, a residue "profile" is built using residues from *L *with which that position structurally aligns. The output allows one to examine commonalities and eccentricities between a target and any number of templates at the structural level, much like a sequence-based profile would allow one to examine standard positional variability.

**Figure 2:**
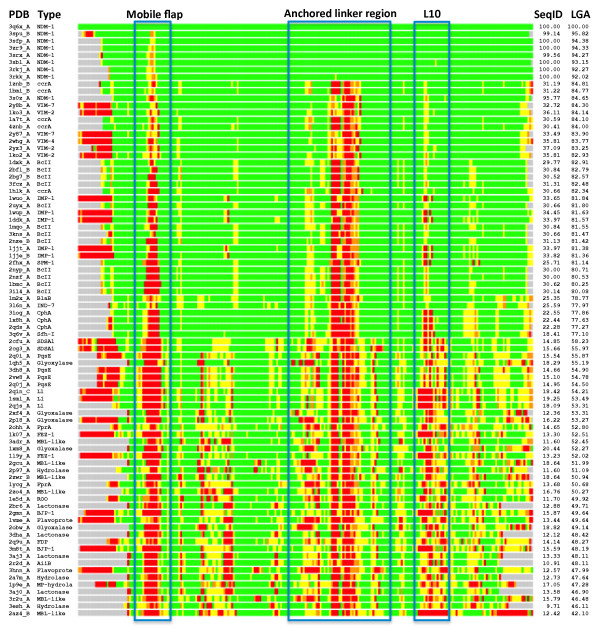
**Heatmap LGA alignments of MBL library against NDM-1**. Parameters for LGA were set using a maximal 4.0 Å distance, under side chain comparison. Coloring was set to > 2 Å (at 2 Å increments) from green to yellow to orange to red to gray. Notable areas of interested are framed using the same ranges as in Figure 1, and include regions of major structural misalignment, such as the L3 mobile flap and the linker region.

To determine structural groupings and gain better insight into both overall and region-specific similarities between MBLs, clustering of the structures was performed using StralCP [34] on a whole-chain level and for two specific local substructures selected for their importance or uniqueness: the active site and the linker region, the latter identified from structural variability analysis to be unique in NDM-1 (see 3.1). Clusters were formed hierarchically using Euclidean distance measurements from *n*-way multiple structural alignments. For whole-chain clusters, terminal regions were trimmed to reduce length bias, while for linker region clustering, corresponding fragments in other MBLs were identified through structural comparisons with a reference structure of NDM-1 (see Figure 2). For example, for the reference ccrA, IMP-1, VIM-2 and NDM-1, the fragments correspond to residues 120-166, 98-143, 138-200 and 141-193, respectively, and include N- and C-terminal anchor sequences that flank the linker region. The active site local environment was delineated using an initial 7.5 Å radius spheres centered at the metal ions. Residues within this radius then formed the core of another, second set of 7.5 Å spheres to produce an additional layer of functional residues. The sphere size was selected in order to capture as much functional information around the local environment as possible, and previous research indicated that distances of 7.5 Å are an upper limit in capturing informative functional properties for clustering purposes [35].

Within the active pocket itself, metal ion distances were measured, and CASTp [36] was used to estimate binding site volumes for B1 MBLs. Because apo and holo forms of IMP-1, VIM-2, ccrA and NDM-1 were available, systematic comparisons of differences in backbone conformation and ligand binding were made. Changes in small molecule binding within IMP-1, VIM-2 and ccrA at the side chain level were compared to NDM-1 for the purpose of classifying NDM-1's functional residue profile using a new pairwise structural comparison service, LGA_pdblist http://proteinmodel.org/AS2TS/LGA_list/.

### Comparisons of critical residues based on structural alignments

Pairwise LGA comparisons were used to examine catalytic and critical residues found in IMP, VIM, ccrA and NDM-1, for the purpose of identifying shared or distinctive conformational changes in the immediate vicinity of metal and ligand binding. Using structural alignments as a scaffold, residue-residue correspondences were generated for B1 MBLs using NDM-1 as a reference. Bound representatives were used for this purpose in order to identify ligand-interacting residues (3q6x_A, 1dd6_A, 1a8t_A, 2yz3_A for NDM-1, IMP-1, ccrA and VIM-2, respectively). Residues specifically examined include those within 4 Å of either the zinc ions or bound ligands. Additionally, residues thought to be critical for other MBL variants based on experimental evidence found in literature, but located outside the active site, were also included and mapped onto NDM-1 for reference.

## Results and discussion

### Overall MBL structure

In the present study, focus was placed on B1 MBLs as a way of generalizing toward emerging, transferable antibiotic resistance genes, such as NDM-1. A global examination of the proteins most closely related to NDM-1 highlighted structural commonalities across B1 MBLs. Comparisons with available NDM-1 structures to the pre-selected MBL-like fold library yielded similar scores, with the closest proteins being representatives from VIM (VIM-2, VIM-4) and ccrA; Figure 2 shows a heatmap of structural alignments of NDM-1 against the preselected MBL library, indicating strong (< 2 Å of Cα-Cα deviation; colored in green) structural concordance in the majority of regions for most other MBLs. The similarity of NDM-1's overall conformation to many other MBLs despite low sequence identity is unsurprising as proteins under the B1 MBL grouping are well known to adopt very similar folds and active regions [14]. However, we note that there are significant regions of divergence, which include the so-called L3 mobile flap, whose motion is associated with MBL ligand binding, and a "linker" loop region commonly found in MBLs. Within VIM-2 this region corresponds to residues 174-186, in IMP it is found between residues 120-129, ccrA residues 142-152 and NDM-1 163-179 (see Figure 1). Structural alignment was generally poor between MBL-types within this region, and it was thus singled out for further analysis.

Beginning at the active site, StralSV analysis of IMP-1, VIM-2, ccrA and NDM-1 type representatives against the preselected MBL library showed well-conserved structural alignment profiles around the di-nuclear zinc binding motif; conservation signals at both the sequence and structure level was strongly evident for the HxHxD zinc binding motif in all four MBLs, even while surrounding residues were generally heterogeneous within the MBL library. Chains that matched this structural region, but did not correspond to the B1 HxHxD, motif were generally B2 MBLs (with an NxHxD motif) or oxidoreductases (*e.g.*, HxExD), illustrating the overall shared conformation of the binding pocket despite variation between actual residues.

**Figure 3:**
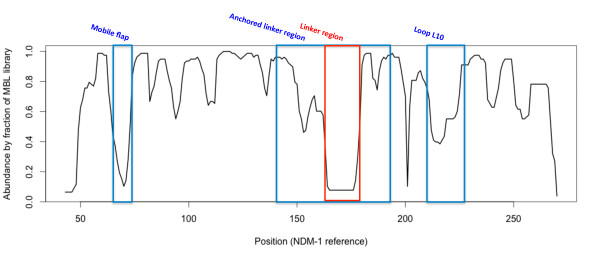
**Structural abundances of NDM-1 based on MBL structural fragment comparisons**. (A) Structural abundance, per residue for NDM-1. The first major decrease in abundance at approximately residue 68 corresponds to the mobile flap found in B1 MBLs. The region of structural disagreement starting at about residue 50 and continuing for 30 residues corresponds to the linker region. The last decrease in abundance, at about residues 210-225 align with a lengthy loop region in other MBLs. The flap, anchored linker region and loop L10 are demarcated using the ranges specified in Figure 2.

Select regions of the B1 MBLs, and indeed the entire library, are less in consensus. As noted previously, the L3 flap region at the entrance to the binding cavity is observed in different local structural conformations in analyzed MBLs. Analysis of the flap region for VIM, IMP and ccrA highlights the divergence between the structures of this flap across B1 MBLs. While known to be functionally important in IMP and ccrA, sequence conservation is limited and structural profiles are heterogeneous due to the region's mobility. Also, whereas IMP and ccrA possess a large side-chain residue (W28 and W49, respectively), VIM lacks the aromatic side-chain and appears one residue shorter than either IMP or ccrA. Phenylalanine (F70) occupies this position in NDM-1, which may serve a similar purpose to that of its analogues in IMP and ccrA. MBL library alignment to NDM-1 using StralSV is shown in Figure 3 as an abundance plot, and represents the fraction of members of the MBL library with structural alignment to NDM-1 over all positions. Across all members of the library, the L3 flap region shows broad disagreement. Graphical illustration of the structural deviations in this region relative to the NDM-1 is shown in Figure 2. Other regions of dissimilarity include the aforementioned linker region and, interestingly, the L10 loop often associated with ligand binding. Most regions otherwise show strong structural agreement, including areas of short misalignment between NDM-1 and other MBLs. This was not surprising, given the conservative fold of MBLs, and even with short insertions, the overall conformation of the proteins would not be expected to diverge greatly [37].

### Structure-based clustering

Structure-based clustering of the entire MBL library showed that MBL folds, including those of the B1 MBLs, group together tightly despite distinct sequence and structure variability in various regions. On the whole-chain level within each subclass, structural differences were generally minimal, and groups of structures cluster cleanly between B1/2/3 MBLs even for the individual B1 types, where ccrA, BcII, VIM, IMP and NDM-1 form distinct branches (with the exception of NDM-1 structure 3s0z_A, which appears to cluster closer to VIM; see Additional file 2). This suggests that while MBLs share a very similar structure, evidenced by the small distances between types on the tree, there are sufficient and consistent differences at the whole chain level that distinguish IMP, VIM, ccrA and other B1 MBLs.

Further structural comparisons across the B1/2/3 MBLs focused on areas of known importance, including the active cavity where zinc ligation occurs. As we were interested in whether this clustering was also evident around the conserved binding cavity, spherical protein substructures with 7.5 Å and centered at the metal ions were extracted, followed by a second layer of 7.5 Å centered around the residues found in the initial step; this two-step approach at spatially defining the active site provided a substructure centered around the binding region encompassing both direct and secondary interacting residues.

**Figure 4:**
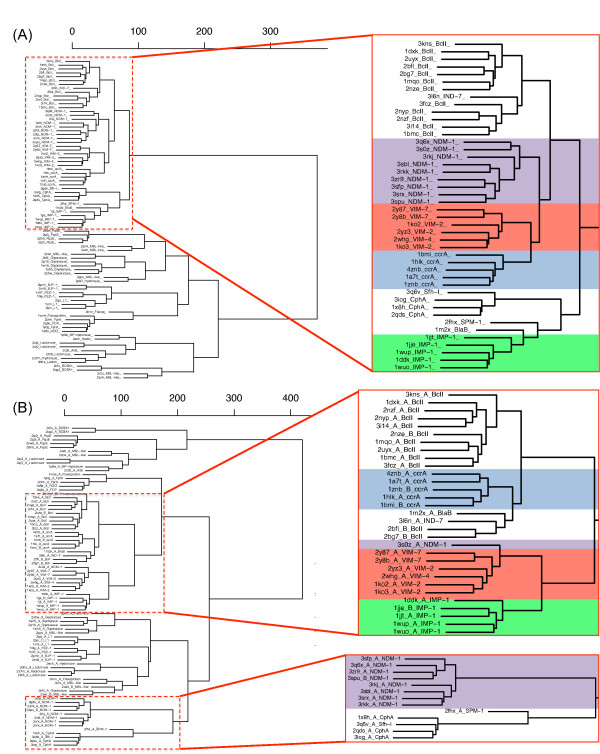
**StralCP dendrograms and clusters for MBL-like proteins**. Structure-based similarity dendrograms based on substructures of the (**A**) active site, and (**B**) linker region, constructed using pairwise average-link Euclidean distances. The subtrees are magnified sections of the dendrograms corresponding to the main branch containing the B1 MBL clusters, as determined *via *StralCP, where coloring correspond to VIM-type (red), IMP-type (green), NDM-1 (purple) and ccrA (blue) structures.

Examination of the clusters formed by extracting the active site and its immediate neighborhood paint a much tighter view of NDM-1, with all instances of the protein clustering tightly, showing that structural features of 3s0z_A outside of the active site are the cause for division (see Figure 4A). VIM- and ccrA-type proteins cluster closely with NDM-1 at the active site. As Yong et al. [2] initially proposed that VIM-2 is a close homolog, the small differences between NDM-1 and VIM are not surprising. However, we note that ccrA, despite being a non-transferrable MBL, is strikingly close in the most critical region to NDM-1, surpassing IMP in active site similarity; indeed, IMP is quite distant from the other transferrable MBLs, indicating relatively strong structural differences between IMP and VIM, NDM-1. Much of this change can be explained by IMP's L10 loop, which is shorter by three residues than ccrA, VIM or NDM-1, a factor believed to affect inhibitor binding [38].

**Figure 5:**
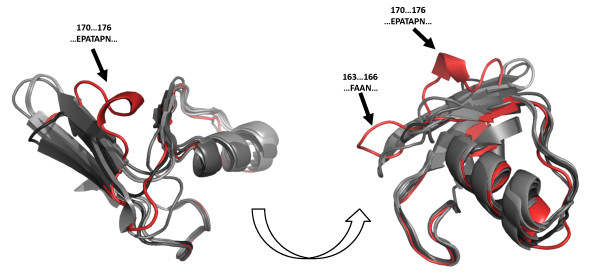
**Visual alignment of the linker region between NDM-1 and other B1 MBLs**. NDM-1 is shown in red, against varying shades of gray for IMP-1, ccrA, VIM-2 and BlaB. N- and C-terminal regions align well across all B1 MBLs, but for NDM-1 the center of the linker region is distal, indicated by arrows and labeled by their positioning and residues within NDM-1. The shown structures correspond to 120-166 in ccrA, 98-143 in IMP-1, 138-200 in VIM-2, 138-200 in BcII and 141-193 in NDM-1; these ranges include N- and C-terminal anchor sequences that flank the linker region and are generally well conserved. Graphics were generated using PyMol [39].

As mentioned earlier, most discordance between B1 MBLs resided within the linker regions of the proteins. As was done for the active cavity, structure-based clustering was done for substructures from the corresponding linker regions. These regions were extracted by using LGA_pdblist to identify N- and C-terminal residues flanking the linker region that had strong structural alignment across the MBL library, followed by structural alignment of only the residues between the flanking regions; results are shown in Figure 4B. Despite the structural variability within this long loop region, most B1 MBLs tended to cluster tightly. However, NDM-1 clustered quite differently from other B1 MBLs, indicative of an entirely novel conformation within its linker region. This deviation, shown in the alignment of the linker region between NDM-1 and other B1 MBLs on Figure 5, results in a significant portion of the NDM-1 linker being exposed. Further manual inspection of structural alignments of NDM-1 to other proteins in the same dendrogram branch (CphA, SPM-1) reveal that the N- and C- terminals align well, but the majority of NDM-1's linker region mismatches.

Within VIM-type proteins, structural alignment reveals that the linker region contains an initial loop approximately five residues longer than the same region in IMP. Incorporating ccrA structures into the pairwise alignment reinforces this five-residue insertion, but also introduces a second insertion in this linker region, producing a loop approximately four residues longer with respect to VIM-type proteins and two residues longer than IMP. Among these three types, IMP represented the structures with both loops short within the linker region, an interesting observation given IMP's aforementioned shorter L10 loop. NDM-1's linker region is extended at the N-terminal, a region where it is most similar to VIM. It then adopts a short helix from positions 170-174, and continues as a loop.

The structural theme found in B1 MBLs is an extended loop pattern, where VIM-type structures possess an initial insertion, followed by a structurally-conserved region approximately five residues in length shared by VIM, IMP and ccrA, and ending with an IMP/ccrA insertion two to four residues in length (see Figure 1). This is in contrast to earlier sequence-based alignments, where the initial VIM insert differs in location, and the later insert toward the C-terminal end is entirely absent [2]. The linker region of NDM-1 is a notable departure from this theme, particularly with the presence of a helix and loop extension. The comparative difference of this region across B1 MBL types suggests the possibility that the linker region is an area of flexibility within MBLs, and that the unusual length and conformation of NDM-1's linker region may confer higher plasticity. Temperature factors of available NDM-1 crystal structures, while generally higher than other stable regions of the structure, were not abnormally high.

### Comparison of MBL pockets and binding changes

**Figure 6:**
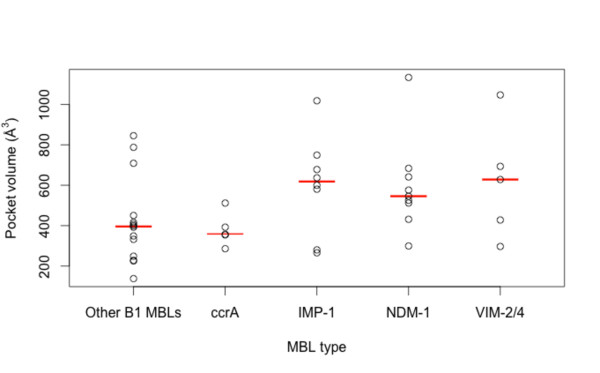
**Estimated pocket volumes of the binding site for ccrA, IMP-1, VIM, NDM-1 and other B1 MBLs**. Boxplots show the distribution of volume for various MBL binding sites; while the plasmid-bourne MBLs (IMP-1, NDM-1, VIM) share similar volume sizes, with fluctuation, ccrA and other B1 MBLs have a distinctly smaller site. Estimates were calculated using CASTp [23]. Data for generating these plots are from Additional file 3.

Estimated calculations of binding site volumes were higher for the plasmid-borne MBLs versus other MBLs (see Figure 6). A larger binding pocket for the B1 MBLs may aid in accommodating a more diverse set of ligands, and on average IMP-1, VIM-2/4 and NDM-1 have similarly-sized pocket volumes. We also find that ccrA, which has close structural homology to transmissible MBLs, has a notably smaller binding pocket site.

**Figure 7:**
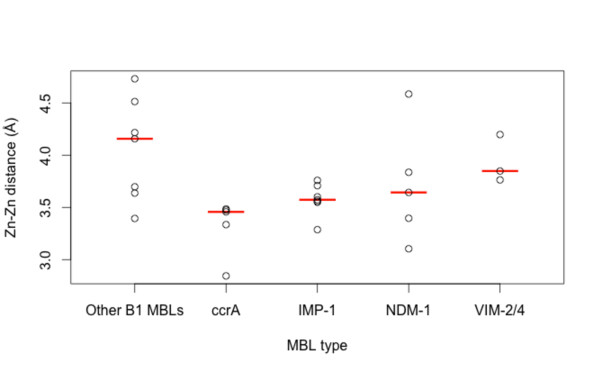
**Estimated metal ion distances for ccrA, IMP-1, VIM, NDM-1 and other B1 MBLs**. Boxplots show the distribution of inter-zinc distances; ccrA has the tightest inter-zinc distance, though this does not appear to be statistically significant when compared to the other types under an adjusted signed rank test. Data for generating these plots are from Additional file 3.

For hydrolytic activity, shallower and tighter zinc ions are associated with more effective catalytic activity [40], and examination of the distances between the MBL zinc ions (for di-zinc species) shows that ccrA ion distances are surprisingly similar to the transferrable MBLs, and IMP-1 and NDM-1 in particular (see Figure 7). Of the plasmid-borne MBLs compared, NDM-1 and IMP appear to have the tightest zinc arrangement, even with the inclusion of a > 4 Å outlier (3q6x_A), whose large inter-zinc distance is likely a result of ampicillin hydrolysis [23]. NDM-1 accommodates both a relatively large pocket volume, similar to VIM, with slightly tighter zinc conformation; these characteristics likely influence its broad binding and catalytic capabilities.

**Figure 8:**
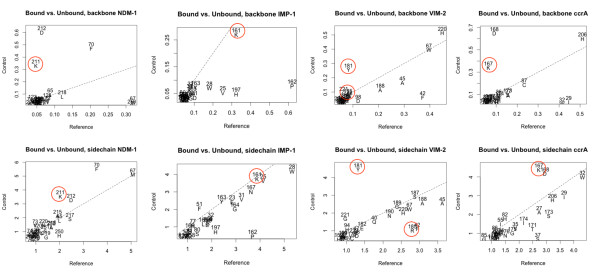
**Structural conformation adjustments within the active sites between bound-unbound structures of NDM-1, IMP-1, VIM-2 and ccrA**. Charts were generated as follows: in each case, a bound reference from one of the four B1 MBL types was selected for examination. The bound reference was compared to other bound B1 MBL structures using LGA (maximal distance of 4 Å), and similarly to unbound B1 MBL structures. Residue shifts within a 4 Å distance of either the bound ligand *or *the dinuclear zincs were drawn on a XY-plot, with the X-axis referring to differences in the bound target and the unbound templates, and Y-axis the bound target against other bound templates, alternatively using NDM-1, IMP-1, VIM-2 and ccrA as a bound representative. The horizontal line shows the line of equal change between bound and unbound comparisons (thus, residues on the top right are residues that deviate in either case). K211/161/167 and R185 (Y181) from NDM-1, IMP-1, ccrA and VIM-2, a ligand binding-associated residue, are highlighted for comparison in red.

Significant backbone and side-chain changes between the bound and unbound states of IMP- and VIM-type proteins, and ccrA and NDM-1 indicated other commonalities and differences among the MBLs. Figure 8 shows these changes between bound and unbound crystal structures around the active site. Specifically, binding across all four MBLs of interest elicits shared, large structural shifts in the flap region: residues M67, F70 in NDM-1 (V25, W28 in IMP-1; I29, W32 in ccrA; F42, A45 in VIM-2) of the mobile flap are shifted during binding via a twist of the loop caused by hydrophobic interactions. Other structural changes are evident in the L10 active site loop, in particular shifts in K211/K161/K167 in NDM-1, IMP-1, and ccrA, respectively, a position that has been associated with polar ligand binding activity in B1 MBL members [23,38,41]. In VIM-2, R185 is believed to play a similar role [42], though in strict residue-residue correspondences from structural alignment, Y181 occupies the position of K211 in VIM-2, and subsequently displays a similar conformational difference between bound and unbound states (Figure 8). Comparison of R185 in VIM-2 shows it undergoes less dramatic a change in conformation, further distinguishing it from the other MBLs (in addition to its relatively shorter length and residue composition). D212/D168 in NDM-1/ccrA, undergoes similar changes in conformation as K211, though the analogous residue in IMP-1, P162, does not.

### Functional and structural residues of interest in B1 MBLs

Structural distinctions between VIM, IMP, ccrA, NDM-1 and BcII (with residue positioning per PDB structures 2yz3_A, 1dd6_A, 1a8t_A, 3q6x_A and 3fcz_A, for context) are identified for residues within 4 Å of either the zinc or ligand binding regions, are shown in Table 1, such as W67 in VIM-2 (2yz3_A numbering; W93 in NDM-1). This residue was determined to be functionally important for VIM-2, was shown *via *mutagenesis to be integral for stability [28] and is bolded in Table 1 due to its colocation (within 4 Å) with the ligand, a mercaptocarboxylate inhibitor [42]. Replacement of this residue in VIM-2 results in decreased ampicillin resistance. 3q6x_A, bound to hydrolyzed ampicillin, indicates the nature of the interaction as a hydrophobic, suggesting a similar antibiotic phenotype [23]. The corresponding residue in ccrA is an isoleucine, which is the second most common residue match using StralSV (see Table 1). IMP-1 possesses a relatively uncommon phenylalanine, though its effect, if any, on enzymatic function has not yet been experimentally characterized. Given the amino acid and similar proximity to an IMP-1 ligand (also a mercaptocarboxylate inhibitor [38]), an analogous effect with W93 in NDM-1 is plausible. Structural variability analysis was also performed using StralSV to identify additional active site conformations and critical residues based on rarity in NDM-1, and several residues were noted to be unique in various parts of the structure (see Additional file 4). Notably, some of these unique residues appear within the active cavity of NDM-1, whose structural corollaries in other B1 MBLs are associated with inhibitor or substrate enzymatic activity. This includes the uncommon residue at the L3 loop (positions 68-72), phenylalanine (F70).

**Table 1:** StralSV profiles of B1 MBL active site, functional residues For each five B1 MBL proteins, residues within 4 Å of either the zinc ions or ligand were identified (red denotes metal coordination residues, while bold denotes those in close proximity to the ligand).

NDM-1 (3q6x_A)	IMP-1 (1dd6_A)	ccrA (1a8t_A)	VIM-2 (2yz3_A)	BcII (3fcz_A)	NDM-1 StralSV profile	Uniqueness, by% shared
**L65**	**E23**	A27	Q40	G59	GDLAEVYCNIQRSWTF	15.5
**M67**	**V25**	**I29**	**F24**	F61	FIDMNHAE	14.1
F70	**W28**	**W32**	A45	-	WFAGKP	24
V73	**V31**	**V35**	**Y47**	V67	VILYPMDKAGQTRHF	46.4
A74	**P32**	**P36**	**P48**	P68	PTDAVISGQLRWCFYHX	7.9
S75	**K33**	S37	S49	S69	SGLAYVKDNEIMRTQHFPC	22.9
N76	H34	N38	N50	S70	NSFTYVAHGQELWIXPMDCRK	26.8
**W93**	F51	I55	**W67**	W87	WIFSTYKPQAVLDGERM	35.8
A116	S73	F78	A90	A112	VIFLASYNWMGTHCQDX	5.5
H120	**H77**	H82	H94	H116	HGVPAIYNLTRWDMQEFS	53.4
**H122**	**H79**	**H84**	**H96**	H118	HGETASNQCKVL	64.4
**Q123**	S80	G85	D97	A119	ALGSDPNFKTYEQVWHIMR	1.6
**D124**	**D81**	**D86**	**D98**	D120	DSNTAKVEIFLPCQGH	73.2
K125	S82	C87	R99	R121	HGRCSANTVFKLYDIME	1.3
Q147	E104	D109	R121	E144	EDLQVKGAHNPSRFTXMYI	7.5
M154	K111	L116	N128	Y167	YKLNMPGF	6
**H189**	**H139**	**H145**	**H159**	H196	HEDKVR	96.9
D202	K152	N158	S172	N215	NRKQADSEHG	5.6
**C208**	**C158**	**C164**	**C178**	C221	DCSXEKMGR	28
**K211**	**K161**	**K167**	Y181	K224	KFYLVHPGR	68.6
D212	P162	D168	E182	S225	SDTEIVA	24.8
A215	-	**T171**	**R185**	A228	ARSTN	78.9
S217	**G164**	S173	S187	D230	DSEG	38.9
L218	L165	I174	A188	L231	LIA	74.8
**G219**	**G166**	**G175**	G189	G232	GL	99.2
**N220**	**N167**	**N176**	**N190**	N233	NKYAPR	85.3
E227	E174	T183	A197	N240	ENKTDIALXPSQ	31.6
Y229	W176	W185	W199	W242	WYMVGLARISTHFN	27.7
G237	K184	K193	Q207	S250	KLRQISEGATVYNC	2.9
S249	S196	G205	G219	S262	GSPAYVDE	24.3
**H250**	**H197**	**H206**	**H220**	H263	HD	99.1
S251	S198	**G207**	G221	G264	GSDNL	15.6
A252	E199	**N208**	L222	E265	EODIALPWVNGY	6.9

These structures were selected because unlike the references structures used to build the MBL library they are ligand bound, with the exception of BcII (3fcz_A). Structural alignments were then used to identify residue-residue correspondences for five B1 MBL proteins for those given residues. For NDM-1, the corresponding StralSV profile is provided, showing several positions in NDM-1, that are either around the binding pocket or are associated with resistance, are rare or uncommon for their corresponding locations in other MBLs, such as 125 K, 123Q, 237 G, 252A, 154 M (full StralSV output is provided in Additional file 4). Note that the residue numbering is according to the PDB structure provided in parentheses: 3q6x_A, 1dd6_A, 1a8t_A, 2yz3_A and 3fcz_A for NDM-1, IMP-1, ccrA, VIM-2 and BcII, respectively. The residue numbering for VIM-2 (2yz3_A) and ccrA (1a8t_A) are different from the residue numbering for the reference VIM-2 and reference ccrA used throughout the rest of this paper

NDM-1 also shares functional residues with MBLs outside the IMP, VIM and ccrA types. Earlier directed evolution studies with BcII indicated several residue changes implicated with resistance [40]. Notably, the glycine to serine change at position 262 in BcII maps to S249 within NDM-1, and S196 in IMP-1 (see Table 1). In NDM-1, as in BcII, S249/S262 forms a hydrogen bond with C208 (3.18 Å)/C221 (3.2 Å), directly affecting the second zinc binding site. This change in BcII is noted to result in increased cephalexin turnover [40]. The complementary mutation within BcII, N70S, is not present in NDM-1, though a similar residue, histidine, is found in IMP-1. Cephalosporin profiles for NDM-1 are most similar to IMP-1, though turnover is slightly better for IMP-1 [2,43], and may imply that a mutation of N76 in NDM-1 to H/S76 may result in more efficient cephalosporin hydrolysis.

**Figure 9:**
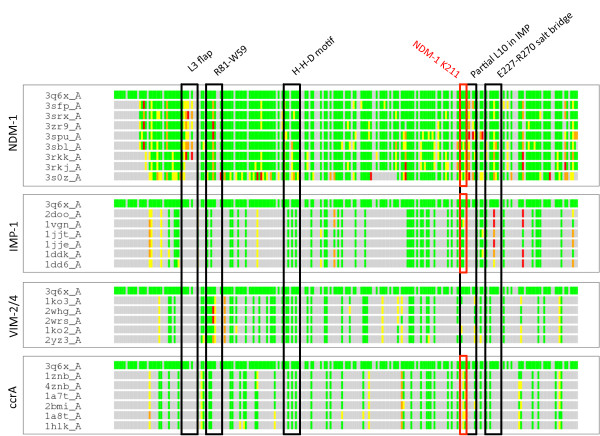
**LGA_pdblist functional side-chain differences between NDM-1 and other B1 MBLs**. Using 3q6x_A as a reference, functional side-chain differences are shown of NDM-1 against IMP-1, VIM-2,4 and ccrA. Measurements are taken only for instances where the residue-residue correspondence for NDM-1 to the other MBLs match, and only then for those with functional sidechains (e.g., alanine, glycine are ignored) [44]. Coloring is from green (< 2Å) to red (< 8Å), with gray indicating no match. Notable regions of B1 MBLs are highlighted, and include the zinc binding region, the L10 ligand-binding loop and the K211 residue noted in Figure 8. Additional annotations show areas where side-chains consistently differ from 3q6x_A and either other NDM-1 structures or other B1 MBLs (e.g., flap region).

Wholesale comparison of these and other possibly critical residues were plotted using LGA_pdblist, permitting a view of deviations between functional side chains of multiple proteins, given a reference. Selection of atom positions for LGA calculation was done using a list of functional ends of protein side-chains, as described in [44]*via *GDC-sc. Use of NDM-1 as a reference against representatives of VIM, IMP and ccrA highlighted areas of inter- and intra-type functional side-chain difference (Figure 9). Between available NDM-1 structures, side-chain positioning is generally in agreement, with the exception of 3s0z_A, which exhibits notable variation not seen in the other representatives (we note that 3s0z_A, an unbound structure, is missing part of the linker region--residues 167-170). Within NDM-1 structures, we observe consistent differences between the bound reference (3q6x_A) and the unbound structures in both R81, the L10 loop and E227 (see annotations on Figure 9). As mentioned in the previous section, IMP, ccrA and NDM-1 also share a lysine (K161 in IMP; K211 in NDM-1) at a residue position associated with mercaptocarboxylate-based inhibition; this residue was highlighted as undergoing conformational changes between bound and unbound states (Figure 8). Side-chain comparisons of this residue using LGA_pdblist show that NDM-1's K211 adopts a functional side-chain orientation closer to that of IMP-1 than ccrA, despite the larger differences between NDM-1 and IMP-1's overall L10 loop. The presence of these residues at similar locations, and notably in comparable conformations, for other MBLs within NDM-1 may contribute to its broad binding profile, whose characteristics are simultaneously close to other B1 MBLs [2].

Close examination of the NDM-1 structures using side-chain deviations from LGA_pdblist as a guide reveal possible electrostatic interactions between R81 and W59, and E227 may form a transient salt bridge with R270. Notably, E227 is located on the turn immediately before the L10 binding loop, and may thus aid modestly in stabilization. While the same glutamic acid is found in IMP-1, there appears no R270 analogue. Comparison of non-covalent interactions between 3q6x_A and NDM-1's unbound representatives using VMD [45] further show that additional salt bridges may form during ligand binding, and that such interactions are more prevalent in NDM-1 than in IMP-1, VIM-2 or ccrA.

Emerging research into the mechanisms of MBL proteins indicate that variation in resistance profiles can be associated with residue changes distant from the active site. Studies of VIM variants and residue-specific changes to members of the IMP type [40,46-48] indicate that locations distant from the active site may affect hydrolytic activity. For example, K215 (aligned to a S172 in VIM-2, > 20 Å distant from the active site) in the recently characterized VIM-19 is associated with improved carbapenem resistance when R228 is also present [48]. The V112A mutation in BcII, similarly distant from the active site, is associated with increased cephalosporin activity, though the association is unclear. As we noted, mapping of resistance-related BcII regions to NDM-1 shows it possess two of four associated hydrolytically beneficial residues. The presence of multiple, fitness-improving residues within NDM-1 found also in critical structural and functional regions of a myriad number of other MBLs suggests incremental and complementary changes in MBL composition, even in regions distant from ligand binding, can have effects on resistance that are difficult to predict.

## Conclusions

We have sought to characterize structural features of members of the B1 MBL proteins most closely related to the recently discovered NDM-1 gene using structural conservation and comparisons of sequence conservation. This has included a survey of the structural features of B1 MBLs from different approaches, including residue variability at specific substructures, clustering varying degrees of structural granularity, and examination of the critical residues of MBLs with an eye toward NDM-1 functionality. While most MBL proteins showed a tightly conserved overall fold structure, structure-based sequence variability methods confirmed the strong structural and sequence conservation at key residues within the active cavity. From this analysis, we find that NDM-1 appears to possess several residues found in variants of IMP, VIM and other MBLs known to confer resistance-like capabilities.

A striking exception to this is the identification of a linker region found within MBLs that appears to vary in structure and length, and is the most divergent and distinguishing structural feature between the IMP, VIM, ccrA and NDM-1 proteins. The identification of this variable linker region within MBLs raised the hypothesis of a distinctive flexible loop; inspection of VIM-2, ccrA and IMP-1 revealed no significant changes in the linker region between apo and holo forms. We identified a marginal difference between the bound and unbound N-terminal ends of the NDM-1 loop on the order of ~1.0-1.5 Å. As the linker region is quite distant from the active site itself, it is unclear if this is a functional shift or an artifact of a possibly more flexible region. Additional study of this region of MBL proteins is necessary to understand how its conformation may affect MBL structure or function.

Deeper knowledge of the structure and mechanism involved in antibiotic resistance in bacteria is highlighted by the continued emergence of transferrable MBLs such as NDM-1. This new enzyme is disturbing for both the speed at which it has spread, its broad capability to bind many types of β-lactams uncharacteristic of other MBLs and its colocation with other resistance-granting genes. Structural alignments of NDM-1 to other B1 MBLs shows that it simultaneously shares critical resistance-associated residues with VIM, IMP, ccrA and even BcII, some of which are distant from the active site. The notion of a structure displaying motifs from multiple protein subclasses is not entirely unknown for B1 MBLs; SPM-1, for example, has structural features found in both B1 and B2 MBL proteins [49]. As others have posited, that this may indicate that while the overall MBL fold structure is critical from a functional standpoint, there is potential for optimization at the residue and substructure level *via *small changes in sequence or conformation [50]; in this light, NDM-1's uniqueness in both composition and structure may serve a multitude of possible function roles, and thus possible targets of further study.

In the future, we hope to expand our computational analysis of these important proteins using ligand screening methods, with the intent to determine residues or structural features that are broadly critical to MBL substrate specificity, thus correlating structure more concretely to phylogenetic profile. The findings described herein provide promising regions for further investigation. Furthermore, experimental follow-up would aid in elucidating the role the linker region may play in MBLs, including NDM-1, with regard to plasticity, function and binding

## Competing interests

Lawrence Livermore National Laboratory holds the patent for LGA (patent #8024127), and has submitted patents for StralCP and StralSV.

## Authors' contributions

EV and AZ acquired the data and conceived the project. AZ, KL and EC conducted the analysis and developed related programs. AZ, KL and EC wrote the manuscript. CZ contributed to discussions and ideas for the project. All authors read, edited and approved the final manuscript.

## Supplementary Material

Additional file 1 **MBL_library.csv--(Comma-seprated values file) Enumeration of MBL folds comprising the comparative analysis library**. This file contains a list of all structures included in the described MBL library (see 2.1), as well as their type classification. Click here for file

Additional file 2 **Whole_chain_clustering--(Portable document format file) Whole-chain clustering of B1 MBL library using StralCP**. This supplemental figure is the whole chain dendrogram for the B1 library, and is depicted in similar form and labeling as Figure 4. Click here for file

Additional file 3 **B1_MBL_active_site_measurements.csv--(Comma-separated values file) Measurements for active sites of selected MBLs**. Each row-wise record of this file contains information regarding the active site of the PDB entry noted in the first column. This data includes: the B1 type, the area and volume of the active site (as estimated by CASTp), an indicator variable associated with the presence of a bound ligand in the active site (1 for a present ligand, 0 otherwise), and the measured metal ion distances for di-zinc MBLs (cases where one or less metal ions are present is designated by a dash). Click here for file

Additional file 4 **3q6x_A_StralSV_w90_5.txt--(Text file) StralSV output for NDM-1 structure 3q6x_A**. This file contains the raw output of the StralSV algorithm run on 3q6x_A using the entire PDB (release 2011/08/02). The header of the file contains structural matches (by region) of various PDB templates to NDM-1, followed by the annotations of the templates. The main body of the file consists of the StralSV output profile, where the first data column is the amino acid; the second column is the position of that amino acid in the profile (starting at 1); the third column is the position of the amino acid in the sequence itself; the fourth column denotes the rank of the amino acid present relative to the structural match profile; the fifth column indicates the percentage of matched structures which have an exact residue-residue correspondence to present amino acid; columns six, seven and eight are the percentages of matched structures which contain the most prevalent, second-most prevalent and third-most prevalent residues, respectively; columns nine and ten are the fraction and number of structural hits; the eleventh column is the StralSV profile itself, sorted by the frequency of the amino acid occupying the position to which the present amino acid aligns; the following columns are indicators for various amino acid categories (see header) and unused measures of conservation. Click here for file
