# Supplementary figures and images for: Computational analysis of pathogen-borne metallo β-lactamases reveals discriminating structural features between B1 types
Source: BMC Res Notes. 2012 Feb 14;5:96. doi: 10.1186/1756-0500-5-96 (PMC3293060; doi:10.1186/1756-0500-5-96)

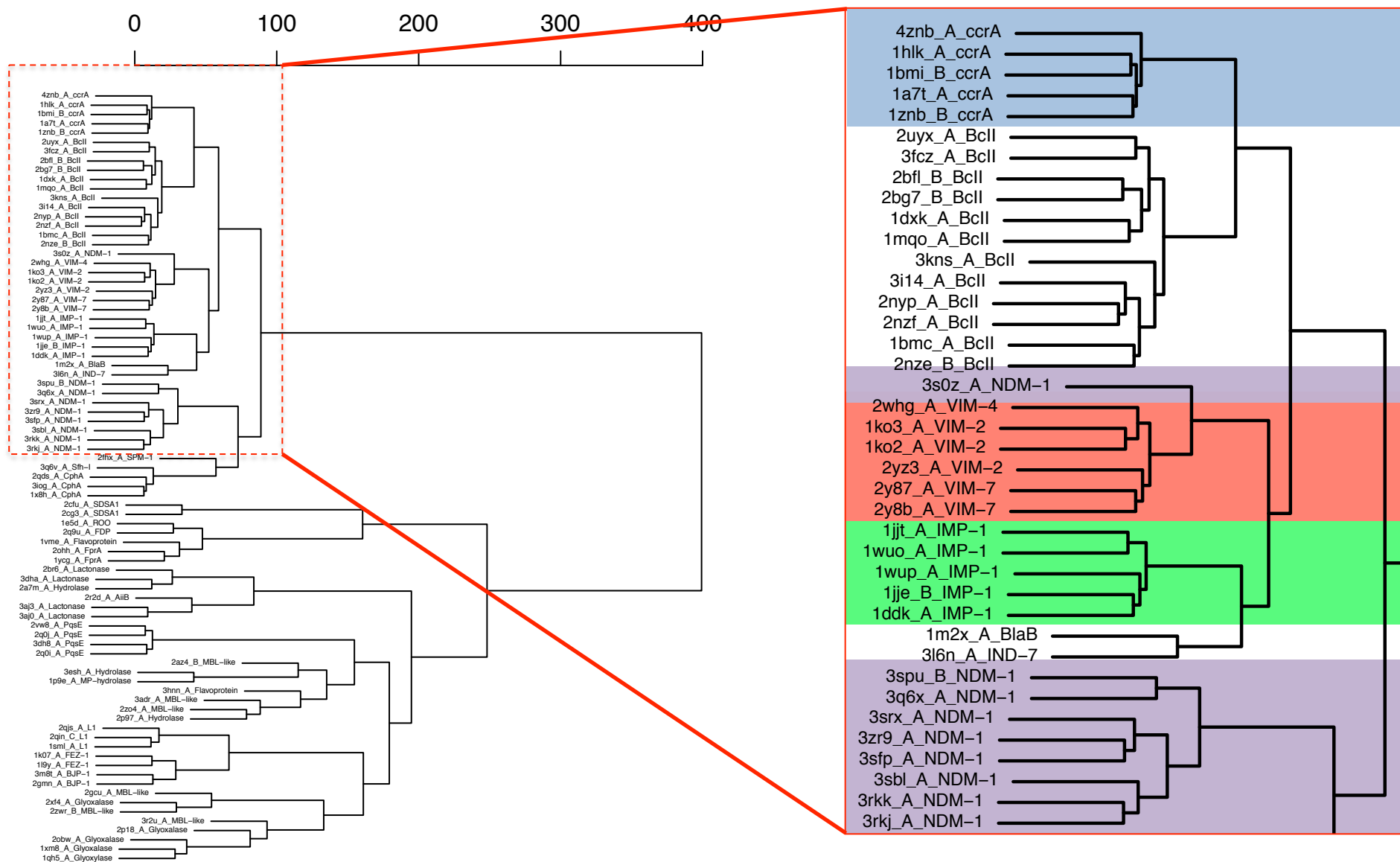

Supplement: Additional file 2 — Whole_chain_clustering--(Portable document format file) Whole-chain clustering of B1 MBL library using StralCP. This supplemental figure is the whole chain dendrogram for the B1 library, and is depicted in similar form and labeling as Figure 4. [file 1756-0500-5-96-S2.PDF]
